# Swarm Optimization-Based Magnetometer Calibration for Personal Handheld Devices

**DOI:** 10.3390/s120912455

**Published:** 2012-09-13

**Authors:** Abdelrahman Ali, Siddharth Siddharth, Zainab Syed, Naser El-Sheimy

**Affiliations:** 1 Schulich School of Engineering, University of Calgary, 2500 University Drive NW, Calgary, AB T2N 1N4, Canada; E-Mails: ssiddhar@ucalgary.ca (S.S.); elsheimy@ucalgary.ca (N.E.-S.); 2 Alastair Ross Technology Centre, Trusted Positioning Inc. (TPI), Calgary, AB T2L 2K7, Canada; E-Mail: zsyed@trustedpositioning.com

**Keywords:** artificial intelligence, systems, measurement, navigation, algorithms, sensor

## Abstract

Inertial Navigation Systems (INS) consist of accelerometers, gyroscopes and a processor that generates position and orientation solutions by integrating the specific forces and rotation rates. In addition to the accelerometers and gyroscopes, magnetometers can be used to derive the user heading based on Earth's magnetic field. Unfortunately, the measurements of the magnetic field obtained with low cost sensors are usually corrupted by several errors, including manufacturing defects and external electro-magnetic fields. Consequently, proper calibration of the magnetometer is required to achieve high accuracy heading measurements. In this paper, a Particle Swarm Optimization (PSO)-based calibration algorithm is presented to estimate the values of the bias and scale factor of low cost magnetometers. The main advantage of this technique is the use of the artificial intelligence which does not need any error modeling or awareness of the nonlinearity. Furthermore, the proposed algorithm can help in the development of Pedestrian Navigation Devices (PNDs) when combined with inertial sensors and GPS/Wi-Fi for indoor navigation and Location Based Services (LBS) applications.

## 1. Introduction

In the recent years, inertial sensors are becoming more popular for navigation in cluttered indoor environments that are challenging for Global Navigation Satellite Systems (GNSS). These Inertial Navigation Systems (INS), consisting of accelerometers, gyroscopes, and a microprocessor, provide position and orientation by integrating the specific forces and rotation rates. Due to the integration process, any errors in the inertial sensor data are accumulated rapidly with time, even with high accuracy sensors. Consequently, regular updates are necessary to provide a drift free position and orientation solution. For updating the position, GNSS signals are utilized, and for heading updates magnetometers may be employed.

For navigation as well as machine control applications, heading information of moving platforms is of paramount importance. Magnetometers based on Anisotropic Magneto-Resistive (AMR) technology depend upon the Earth's Magnetic Field (EMF) from which the heading information can be derived. The ubiquitous nature of EMF makes these sensors available in airplanes, vehicles, ships, and they are now being explored in hand-held devices. In order to improve the robustness of the heading solution, an optimal fusion of these sensors is justifiable [1]. This depends upon the cost, accuracy, and type of application at hand.

In addition to the accelerometers and gyroscopes, magnetometers can be used to derive the user heading, by sensing the Earth's magnetic field. The magnetometer employed in this research is devoid of any drifts as observed from the Allan variance plot of the long-term static data. This, therefore, gives us the opportunity to compensate any drift from the gyroscope. Hence, we can use the magnetometer over extended periods of time, especially in magnetically stable environments. The stability of an environment is characterized by low ferrous objects, or power-line nearby the magnetometer. In dense indoor areas, the problem of navigating a user becomes even more challenging, due to the proximity to metallic objects and walls, supported by ferrous pillars.

In most of the early research, the calibration of magnetometers either accomplished in the heading domain [2] or in, the magnetic field domain [3,4]. The advantage of applying the calibration algorithm in the magnetic field domain is convincing, as we do not have to depend on the heading of the sensor prior to calibration. For a given region, the Earth's total magnetic field is constant and its value can be obtained from the International Geomagnetic Reference Field (IGRF) model. This becomes a basis for developing a mathematical model for sensor calibration [5]. Magnetic field sensors are classified on the basis of the technology adopted in their manufacturing into the following types [6]:

- Anisotropic Magneto-Resistive (AMR)
- Hall Effect Sensors
- Magneto-elastic Sensors
- Fluxgate Sensors
- Mechanical Magnetic Sensors

The sensor used in this research is based on the AMR technology and will be discussed briefly. The discussion of all other technologies is beyond the scope of this research. The magnetometer adopted in this research is a tri-axial sensor which converts any external magnetic field sensed by the sensitive axis directions to a differential voltage output. The AMR sensors are made up of thin-film Perm-alloy (nickel-iron) material, patterned as a resistive strip. When a magnetic field is applied, the element being sensitive to such fields undergoes a resistivity change, leading to a change in resistance. This disturbs the balance of the Wheatstone bridge configuration leading to a differential change in voltage output, which is proportional to the differential change in incident magnetic field. Because the output is proportional to the one-dimensional axis (principle of anisotropy) and its magnitude, additional sensor placed at orthogonal directions permit measurement of arbitrary field directions (HMC5843 data sheet).

Apart from having directionality and high sensitivity, these sensors also have a unique property called “flipping” action, associated with dipole switching due to internal magnetization in the presence of oscillating external fields. This behaviour may be exploited in eliminating undesirable DC-offsets in the presence of weak magnetic fields [6]. Different reasons are listed to use the PSO based technique over well-known estimators, such as Extended Kalman Filter (EKF) where the drawbacks of such techniques can be summarized as below [5]:

- *A priori* knowledge of initial states.
- Inaccurate knowledge of noise statistics (Process Noise/state Covariance).
- Matrix implementation, especially, inversion operation which may lead increased computation time and leads to singularity.
- Higher heading initialization uncertainty.

Artificial Intelligence (AI)-based algorithms are considered as practical tools for nonlinear optimization problems [7], as these algorithms do not require that the objective function be differentiable and continuous. Different approaches are implemented based on AI such as Artificial Neural Network (ANN), Genetic Algorithms (GA), and Swarm Intelligence (SI). SI is the property of a system whereby the collective behaviors of (unsophisticated) agents interacting locally with their environment cause coherent functional global patterns to emerge. SI provides a basis with which it is possible to explore distributed problem solving without centralized control or the provision of a global model [8,9]. Anti-Colony Optimization (ACO), Bees Algorithm, and Particle Swarm Optimization are different approaches or versions of SI, which are implemented and explained in detail in the literature. PSO is one of the modern heuristic algorithms [10] and can be applied to nonlinear optimization problems [11]. It has been developed through simulation of simplified social models. PSO has gained wide recognition due to its ability to provide solutions efficiently, requiring only minimal implementation effort.

In this paper we introduce a PSO-based technique for calibration of magnetometers by estimating the values of the low cost magnetometer bias and scale factor. We introduce a robust parameter estimation technique, which has its origin in AI. The method referred to here as PSO is best suited for problems of non-linear and non-Gaussian nature. This consideration also becomes important since we don't know the nature of external fields corrupting the magnetometer's signal. PSO is a better choice to circumvent all these difficulties, and is extensively employed to solve complicated design optimization problems as it can handle both discrete and continuous variables as well as nonlinear objective and constrained functions without the computation of a gradient [12]. Three bias and three scale factor terms corresponding to each axis of the tri-axial magnetometer are estimated, which constitute the six elements of the state vector.

Section 2 of the paper provides the mathematical background for calibration, while Section 3 has a brief discussion of particle swarm optimization technique. Section 4 describes the proposed estimator algorithm adopted in magnetometer calibration. The calibration test results with real magnetometer data are given in Section 5. The paper ends with a conclusion in Section 6.

## 2. Constrained Calibration Approach

Based on the Earth's magnetic field, the formulation can be stated by the following mathematical model [5]:

(1) B=AH+b+ɛ

Equation (1) can be rewritten in the form:

(2) $$H=A^{-1}(B-b-ɛ)$$

where:

- ***H*** is 3 × 1 estimated EMF vector,
- ***B*** is 3 × 1 measured magnetic field vector, magnetometer readings, B = [*B_x_ B_y_ B_z_*]*^T^*,
- ***A*** is 3 × 3 scale factor matrix where, *A* = *diag*(*a_x_*, *a_y_*, *a_z_*),
- ***b*** is 3 × 1 bias vector, where *b* = [*b_x_ b_y_ b_z_*]*^T^* and
- ***ε*** is 3 × 1 noise vector, ***ε*** = [***ε*** *_x_* ***ε*** *_y_* ***ε*** *_z_*]*^T^*

To simplify the mathematical formulation we can ignore the white noise which is not part of the model used for calibration parameters in the estimation process, in this case, Equation (2) can be rewritten as:

(3) $$H=A^{-1}(B-b)$$

The bias and scale factor are estimated subject to the following objective function:

(4) $$H_{m}^{2}-{\left\|H\right\|}^{2}=H_{m}^{2}-H^{T}H=0$$

where H_m_ is the true, reference, magnitude of the Earth's magnetic field in a given geographical location (can be obtained from the IGRF model). Every five years, a group called the International Association Geomagnetism and Aeronomy (IAGA) revise the IGRF parameters. The user is required to input the latitude, longitude and height of the place where the Earth's magnetic field intensity is sought. The 11th generation IGRF accepts the years in between 1900–2020. The accuracy of the estimated Earth's magnetic field is claimed by the IAGA to be 1 nT (0.01 mGauss).

## 3. Particle Swarm Optimization

Bird flocks, fish schools, and animal herds are examples of natural systems where an organized behaviour is successful in producing impressive, collision-free, and synchronized movements [10]. In these natural systems, the behaviour of each group member is based on simple inherent responses. SI is mainly inspired by such kinds of animal and natural systems. Although SI is still in its infancy compared to other paradigms in artificial intelligence, it offers an attractive new research field.

A promising performance is shown by swarm-based algorithms, being efficient, robust and very simple to implement [13]. One of the most interesting research areas within computational swarm intelligence is the PSO which was developed based on the concepts and rules of socially organized populations in Nature, such as bird flocks, fish schools, and animal herds. The swarm consists of a group of individual agents called particles. Each particle follows a simple behaviour to achieve best performance by following the best of the group. PSO is a population based stochastic optimization technique, developed by Eberhart and Kennedy in 1995 [14]. They claimed that searching for a food source is similar to finding a solution for a common research goal [15]. In comparison with other AI optimization techniques, the power of PSO lies in its simplicity of implementation. The performance of different optimization techniques used in industry today, along with their computational efficiency, clearly indicates that PSO performed better than other algorithms in terms of success rate, solution quality, and convergence speed [16]. It can be applied to solve various functional optimization problems. Moreover, PSO can work in cases of non-differentiable transfer functions where no error information is available [17].

**Figure 1.**
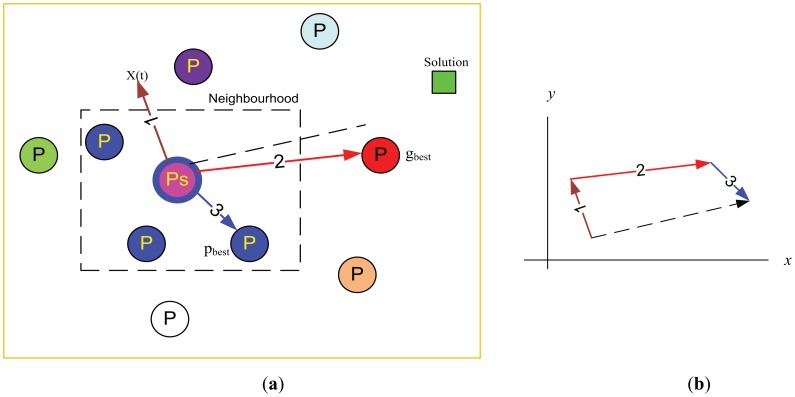
**(a)** Particle local and global best. **(b)** Particle vector components.

The PSO technique employs a set of feasible solutions called a “swarm of particles” that are populated in the search space with initial random positions and velocities as shown in Figure 1(a). At any particular instant, each particle has its own position and velocity [18]. Each particle is trying to get its own solution for the problem in the search space to target the optimal “solution”. All particles have fitness values which are evaluated by the cost or fitness function to be optimized, and have update values, velocities, which control the movement of the particles. PSO is initialized with a group of random particles (solutions) and then searches for optima by updating generations. The algorithm is iterative and the locations will change at each time step. In addition, each particle will record the location of its ‘best position’. In every iteration, the particle (P) is updated by two best values. The first one is the best solution (fitness) it has achieved so far, where the fitness values are stored, during the process up to current iteration and it is local best “particle” and called p_best_ (*p_i_*). The other best value is the global best and called g_best_ (*p_g_*) which is the position that has been found to be the fittest so far from among all the particles in the population [19–23]. After finding the two best values, the algorithm then updates the position of each particle iteratively by following Equations (5) and (6) until the process is terminated. Figure 1(b) shows the visualization of the PSO vector components during the update process. The updated position is resulted from the summation of the basic vectors of the current position, local best vector, and global best vector [24].

For a swarm of *N* particles and search space of dimension *D*, define the *i^th^* position and velocity of the particle as *x_i_* = *(x_i1_, x_i2_*, …, *x_iD_)* and *v_i_* = *(v_i1_, v_i2_*, …, *v_iD_)* respectively. The PSO algorithm can be performed by the following Equations:

(5) $$v_{i}^{k}=w.v_{i}^{k-1}+c_{1}r_{i1}^{k-1}(p_{i}^{k-1}-x_{i}^{k-1})+c_{2}r_{i2}^{k-1}(p_{g}^{k-1}-x_{i}^{k-1})$$

(6) $$x_{i}^{k}=x_{i}^{k-1}+v_{i}^{k}$$

where:

- *k* is the index of the current, new, iteration and *k-1* refers to the previous, old, iteration.
- *i* = *1, 2*, …,*N* where *N* is the size of the population, number of particles.
- *c_1_* and *c_2_* are acceleration coefficients, usually *c_1_* = *c_2_* = *2*.
*r_i1_* and *r_i2_* are random numbers uniformly distributed within the range [0, 1].
- *w* is inertial weight factor, and the bigger the value of *w*, the wider is the search range.

Equation (5) is used to to estimate the update of change in position, velocity, of the *i^th^* particle while Equation 6 provides the new position. During the process we used fixed values for *w*, c_1_, and c_2_ to be 1, 2, and 2 respectively:

(7) $$\Delta H=H_{m}^{2}-H^{T}H$$

(8) $$\text{Fit}\_\text{Value}=\sqrt{\sum {\left(\Delta H\right)}^{2}}$$

where Δ*H* is the error magnitude of total magnetic field. The performance of each particle is measured according to a fitness function, which is problem-dependent. In optimization problems, the fitness function is usually identical with the objective function under consideration. Equation (7) shows the used fitness function which it is the difference (error) between the estimated total magnetic field and the reference value. The reference value is 170 mGauss in the case of 2-D calibration and 560 mGauss for the 3-D calibration case. The fitness value is computed in Equation (8) as the square root of the summation of the squared error.

**Figure 2.**
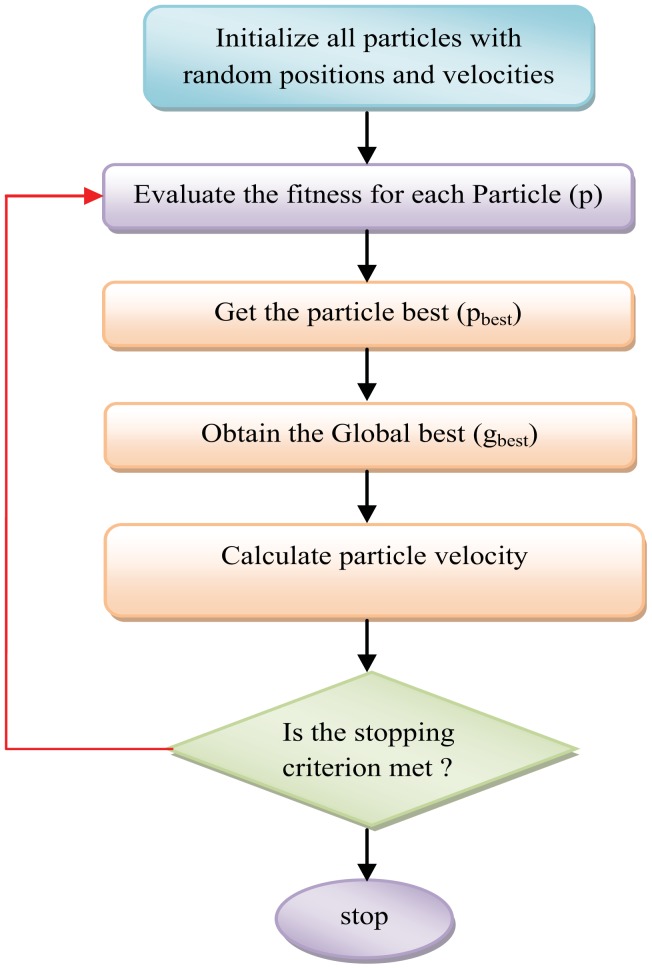
The basic PSO algorithm.

The algorithm re-evaluates all particles' locations after every iteration and gets the new best values. To find the optimum value, a recurring searching process is done until the maximum iteration number is reached or the minimum error condition is achieved. The PSO general computational steps are shown as in Figure 2.

## 4. PSO Based Calibration Technique

### 4.1. Basic PSO Based Calibration Algorithm

The proposed algorithm is used to estimate the bias (*b*) and scale factor (*SF*) of the magnetometer by minimizing the difference between the measured magnetic field and true one. It improves the heading results because it exploits the fact that the incorrect heading estimates due to the magnetometer biases, scale factors and declination angles have a relationship with the true heading. The PSO algorithm is used to estimate the required parameters for calibration.

As mentioned in Section 2 and by substituting from Equation (3) in Equation (4), the difference between the true Earth magnetic field and the measured one can be rewritten as:

(9) $${\left(A^{-1}(B-b)\right)}^{T}(A^{-1}(B-b))=H_{m}^{2}$$

(10) $$\begin{array}{c} {\left(A^{-1}(B-b)\right)}^{T}(A^{-1}(B-b))-H_{m}^{2}=0\\ {\left(B-b\right)}^{T}{\left(A^{-1}\right)}^{T}A^{-1}(B-b)-H_{m}^{2}=0\\ {\left(B-b\right)}^{T}\lambda (B-b)-H_{m}^{2}=0\\ B^{T}\lambda B+uB-K=0 \end{array}$$

where *λ* = (*A*^−1^)*^T^* A^−1^, *u* = −2*b^T^ λ*, $K=b^{T}\lambda b-H_{m}^{2}$.

The value of scale factor matrix A is evaluated as the diagonal matrix of the scale factor where *A* = *diag(SF)*:

$$A=\left[\begin{array}{ccc} a_{x} & 0 & 0\\ 0 & a_{y} & 0\\ 0 & 0 & a_{z} \end{array}\right]$$

The main objective of proposed technique is to estimate the values of the scale factor and bias respectively according to Equation (8) where *SF* = [*a_x_*, *a_y_*, *a_z_*]*^T^*, and *b* = [*b_x_*, *b_y_*, *b_z_*]*^T^*.

### 4.2. The Range of Interest Selection Technique (RIST)

**Figure 3.**
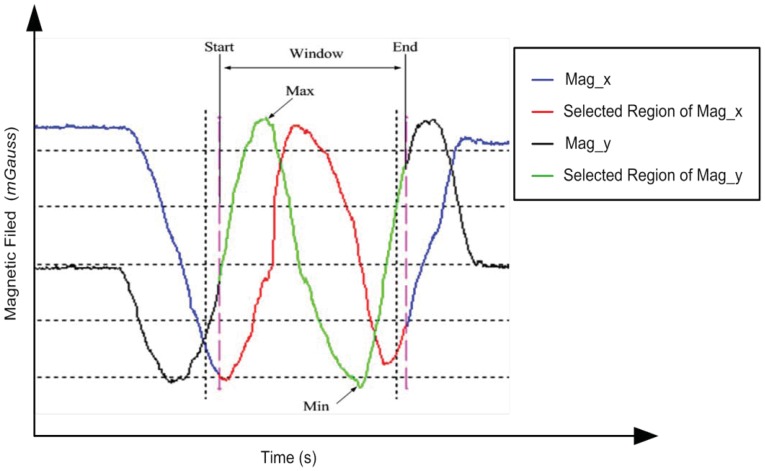
The range of interest selection technique principles.

In all previous work on calibration, the selection of a range of interest of the measurements is not taken in account. This might lead to inefficient calibration and increased time complexity. In order to make the calibration procedure more efficient, the appropriate range of the signal from the entire dataset is extracted and processed. The RIST is used to select the most effective part of the raw data which will be used during the calibration process. The proposed technique is searching for the maximum change in the magnetic field for each axis and gets the interval in between. The algorithm receives the overall raw measurements and returns the start and end indices of the nominated interval as shown in Figure 3. The selection operation is based on detecting the maximum and the minimum signal amplitude in the raw measurements. Based on the selected indices, the range of interested is extracted and passed to the calibration algorithm to get the estimated values for *SF* and *b*. The pseudo-code for the RIST algorithm is given in Appendix 1.

RIST is important for the real time calibration which is a typical process for most of the compass calibration in a personal navigation devices or smart phones. In doing so, the calibration algorithm becomes fast, and therefore, fits the real time requirements. In order to improve upon this methodology, outlier detection may be implemented to improve the results by distinguishing between signal max/min arising out of rotation and external disturbance or noise. The trust in selecting the peak values may further be enhanced by observing the signal pattern of other sensors. These sensors might be found in the same device (e.g., gyroscopes and accelerometers), which attains a specific pattern during rotation motion as the calibration of the magnetometer requires a full rotation of the sensor on a (horizontal/vertical) plane.

**Figure 4.**
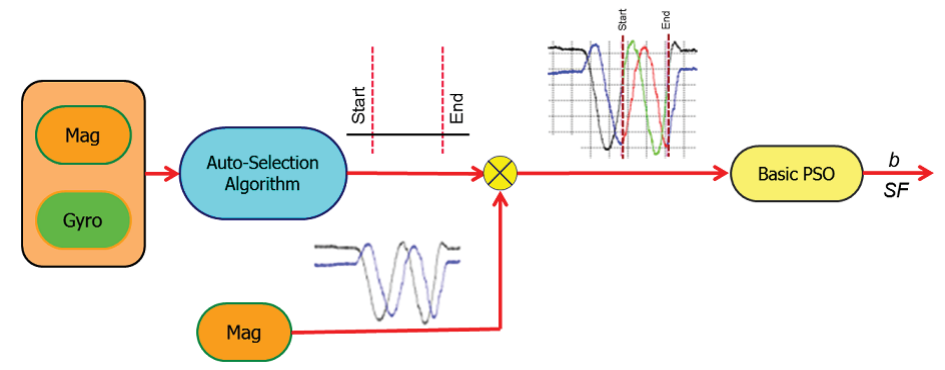
RIST algorithm.

Figure 4 shows the first stage of the proposed calibration scheme. The magnetometers and gyroscopes measurements are entered to the RIST to be trimmed. The output of the auto-selection algorithm is the start and the end of the selected range of the magnetometer measurement.

The embedding of RIST into the scheme leads to less samples needing to be processed during the calibration step. As a result, the total time of the calibration process is decreased which leads to power savings for consumer devices.

### 4.3. Modified PSO Technique (MPSOT)

**Figure 5.**
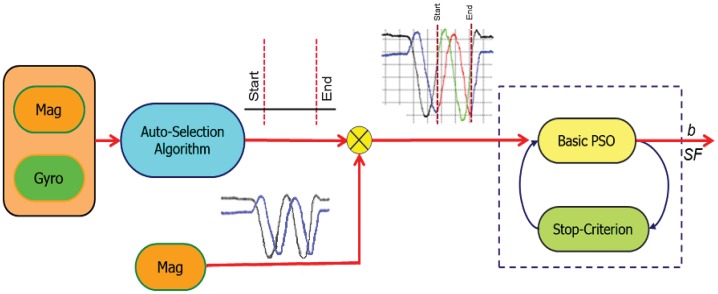
MPSO based calibration algorithm.

The PSO algorithm is based on an iterative process to reach the optimum solution. Through this algorithm, an iterative process is conducted to estimate *SF* and *b*. Over the iterations these values converge to the best values and the process is terminated. The purpose of MPSOT is to create new criteria to stop the estimation process. The pseudo-code for the MPSOT is given in Appendix 2. As shown in Figure 5, the basic PSO algorithm is modified by creating termination criteria for the calibration process to reduce the processing time. The stop criteria take three different levels:

- Maximum number of iterations.
- Hit a minimum error value.
- Change in bias and scale factor values becomes less than a threshold of 0.01 for consecutive iterations.

## 5. Results

To assess the overall performance for the proposed new calibration technique, a group of field tests were conducted at the University of Calgary campus. The tests include collecting data in static, walking, indoors and outdoors mode of operations. The collected data are the raw sensor readings from the Honeywell 3-axis magnetometer (HMC5843). The data rate of the magnetometer readings is 40 Hz with 25 ms between two consecutive epochs. For the 2-D calibration, the magnetometer was rotated 360° in the horizontal plane and then the heading was computed using the estimated SF and b. The 3-D test was conducted by rotating the device about the vertical axis followed by the horizontal axis rotation. In all tests, the algorithm successfully converges to a good estimate of the SF and b values and showed improvement in terms of heading results after the calibration.

The results are described below where the basic PSO algorithm is first applied using the entire dataset of the magnetometer measurements in the parameters estimation process. With the basic PSO there is no modification in the core of the PSO algorithm. Secondly, the effect of applying the RIST is illustrated where a range of interest of the dataset is selected to be used instead of the entire dataset as described in Section 4.2. Finally, the MPSOT is used where a significant change is applied to the core of the basic PSO algorithm.

### 5.1. 2-D Calibration

2-D calibration tests are done by holding the PNS in the horizontal plane. The total magnetic field is calculated in the horizontal frame, *x* and *y*. The reference value of the total Earth's magnetic is 170 mGauss. We used the measurements for magnetometers *x* and *y*. Tests were performed in the multi-sensors lab at the University of Calgary. Tests include two 360 degree turns about the z-axis using a rotation table to be sure that the device is held in the horizontal plane. *SF* and *b* values are estimated by passing the magnetometer readings to the basic PSO algorithm.

#### 5.1.1. Basic PSO Results

**Figure 6.**
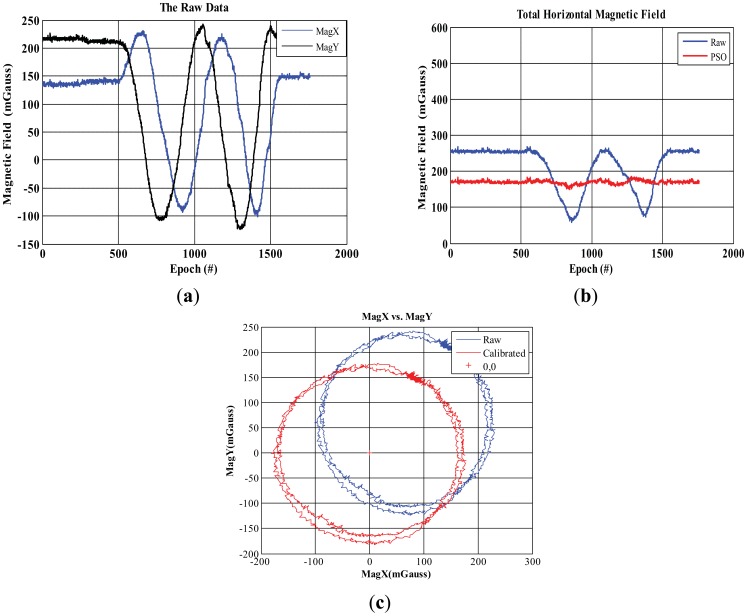
Basic PSO calibration. (**a**) Raw magnetic field. (**b**) Horizontal raw and PSO calibrated magnetic field. (**c**) 2-D calibration for adjusted magnetic field.

Figure 6 shows the results for the basic PSO where “Raw” and “PSO” terms refer to the calculated magnetic field based on the raw measurements and the calibrated one respectively. Figure 6(a) shows the raw magnetic field in the *x* and *y* directions while the total horizontal magnetic field is shown in Figure 6(b). The calibrated readings show the constancy of the estimated magnetic field, which closer to the true EMF. Figure 6(c) shows the resulted track after calibration where the two 360 degrees turns about z-axis are calibrated and adjusted around the origin with the shape of circles based on the values estimated by the PSO algorithm.

#### 5.1.2. RIST Results

**Figure 7.**
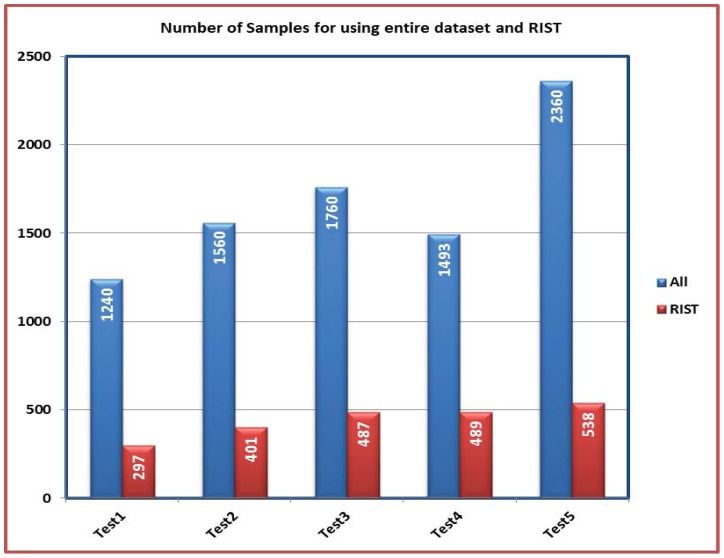
A comparison between the numbers of samples applied for magnetometer calibration in case of using the entire dataset and RIST in 2-D calibration.

In this section, the impact of applying RIST in reducing the time required to estimate *SF* and *b* values is observed. The number of samples is compared in the cases of using the entire dataset and the range of interest. The result is Figure 7 where the total number of samples is decreased for all tests. For example; the number of measurement samples which used in Test1 is 1,240 whoever applying the RIST reduces the number to 279 samples. As a result, this decrease leads to decrease the required time to perform the calibration process. Although less information is fed to the PSO algorithm, the accuracy of the results hasn't been affected as indicated in Table 1(a,b) where the values of *SF* and *b* are close for all tests at both cases. This indicates that the results from the PSO with the entire dataset and with range of interest almost have the same behaviour.

#### 5.1.3. MPSOT Results

**Figure 8.**
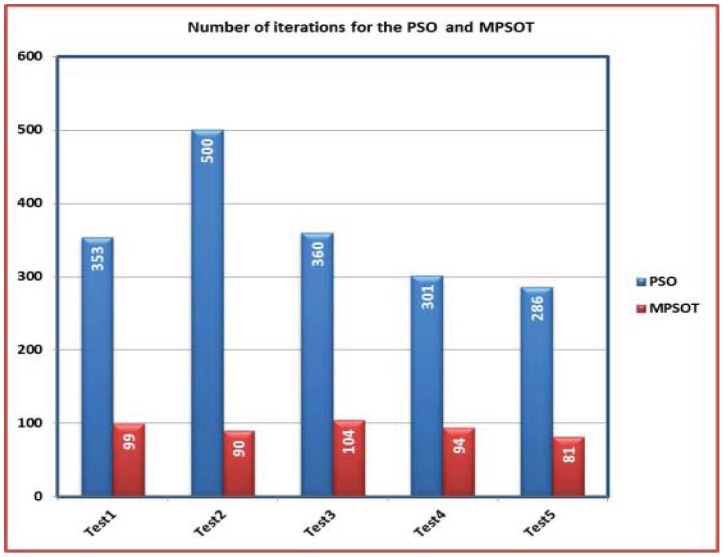
A comparison between the numbers of iterations in case of using the basic PSO and MPSOT in 2-D calibration.

To show the final impact of the proposed algorithm, both RIST and MPSOT are fused and applied. In such scenario, the entire dataset is applied to RIST to produce the range of interest of the dataset. Thereafter, the MPSOT is applied to reduce the number of iterations required by the algorithm to converge. Figure 8 shows a comparison between the number of iteration, which the algorithm consumes to converge in the case of using basic PSO, and its modified version—MPSOT. The comparison shows that the number of iterations is decreased to third in most cases when MPSOT is applied. Without doubt, the values of SF and b haven't been affected as illustrated in Table 2(a,b).

### 5.2. 3-D Calibration

To examine the performance of the proposed technique in the 3-D calibration, six different tests are conducted. During the 3-D tests, the device is moved freely in the space. Actually, the 3-D calibration is more convenient for the user where the device shouldn't be held in the horizontal plane. The total magnetic field is calculated where the reference of the total Earth's magnetic is 560 mGauss. The algorithm receives data from the 3-axis magnetometer. The results are discussed below where it conforms to the previous discussion on the performance of the 2-D calibration.

#### 5.2.1. RIST Results

**Figure 9.**
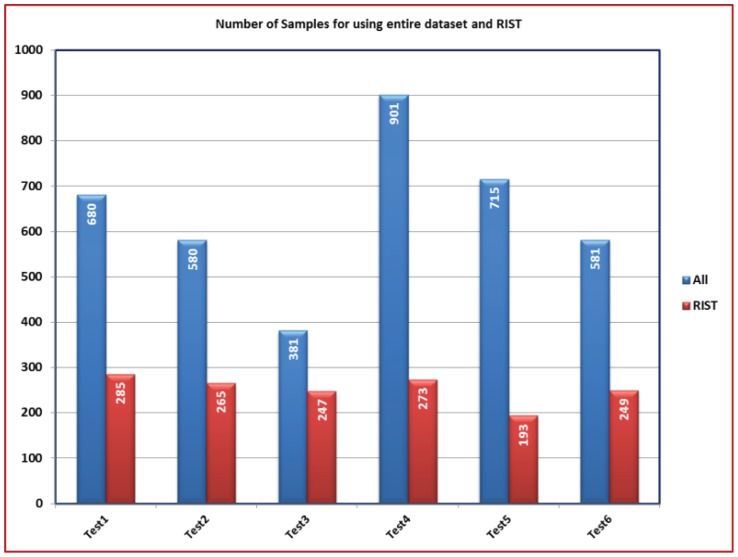
A comparison between the numbers of samples applied for magnetometer calibration in case of using the entire dataset and RIST in 3-D calibration.

Again, Figure 9 shows the comparison between the number of samples in both cases of using the entire dataset and the region of interest. Obviously, Table 3(a,b) shows there is no big change in the values of the *SF* and *b*. These results show the validity of the proposed calibration technique even at a lower number of observations. Like the previous case in 2-D calibration, comparing the results of the RIST shows the benefit of the technique proposed for calibrating the magnetometer.

#### 5.2.2. MPSOT Results

**Figure 10.**
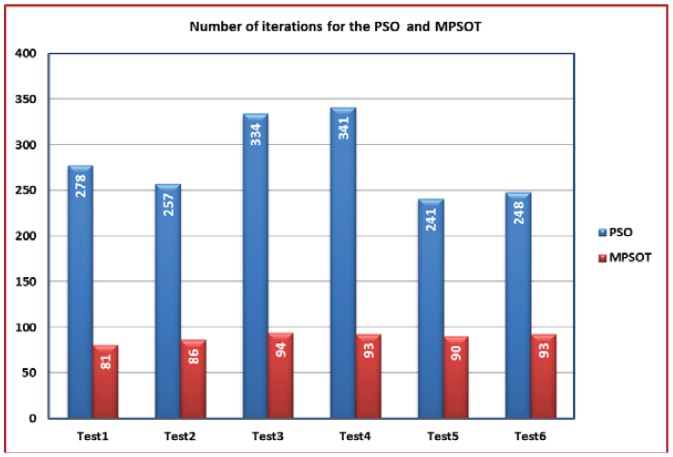
A comparison between the numbers of iterations in case of using the basic PSO and MPSOT in 3-D calibration.

Figure 10 shows the comparison between the number of iterations for the basic PSO and the MPSOT. Table 4(a,b) shows the different values of *SF* and *b* for both algorithms where the values are close. These results show that the MPSOT reduces the time required for the calibration process like the previous case in 2-D calibration.

## 6. Conclusions

In this paper, a PSO based calibration algorithm to estimate the values of the bias and scale factor for a low cost magnetometer is presented. In our approach, the selection of the effective range from the entire dataset is made automatic. In addition, a significant change in the core of the basic PSO algorithm is proposed to reduce the required time for the calibration process. The proposed technique has many advantages over any other conventional method such as the use of the artificial intelligence, which does not need any error modeling or awareness of the nonlinearity. The use of the RIST reduces the processing time to 40% of the time for the entire dataset while MPSOT improves the overall processing time to 35% of the time consumed by the basic PSO. The estimated bias and scale factor values from the proposed algorithm improve the overall performance of the calibration process with regard to the accuracy and time complexity. This technique would help decrease the heading errors of the users in pedestrian navigation. It can also help in the development of Pedestrian Navigation Devices (PNDs) when combined with the INS and the available RF signals, especially in indoors environments. Clearly, the fusion of the RIST and MPSOT techniques decreased the required time for the calibration process, extending the opportunity to apply the proposed algorithm in real-time applications.

## Figures and Tables

**Table 1.** Magnetometer parameters values resulted from using the entire dataset and applying RIST in 2-D calibration. (**a**) Scale factor. (**b**) Bias.

	**X Scale Factor**		**Y Scale Factor**				**X Bias**		**Y Bias**	
	**All**	**RIST**	**All**	**RIST**			**All**	**RIST**	**All**	**RIST**
**Test1**	0.873	0.866	0.998	0.973		**Test1**	87.279	87.419	54.190	50.008
**Test2**	0.601	0.598	0.655	0.649		**Test2**	33.490	32.674	42.887	42.193
**Test3**	0.925	0.913	1.018	1.050		**Test3**	66.016	63.461	61.332	58.556
**Test4**	2.498	2.535	2.720	2.773		**Test4**	36.912	50.207	42.229	51.725
**Test5**	0.791	0.793	0.872	0.869		**Test5**	19.483	19.986	42.121	43.142
(**a**)						(**b**)				

**Table 2.** Magnetometers parameters resulted from using the basic PSO and MPSOT in 2-D calibration. (**a**) Scale factor. (**b**) Bias.

	**X Scale Factor**		**Y Scale Factor**				**X Bias**		**Y Bias**	
	**PSO**	**MPSOT**	**PSO**	**MPSOT**			**PSO**	**MPSOT**	**PSO**	**MPSOT**
**Test1**	0.858	0.863	0.982	1.027		**Test1**	85.615	86.025	50.381	46.695
**Test2**	0.597	0.602	0.649	0.629		**Test2**	32.659	29.96	42.003	43.227
**Test3**	0.913	0.911	1.05	1.049		**Test3**	63.563	62.955	58.489	58.503
**Test4**	2.534	2.535	2.773	2.773		**Test4**	50.356	50.162	51.822	51.59
**Test5**	0.787	0.775	0.856	0.859		**Test5**	18.75	16.295	40.415	47.76
(**a**)						(**b**)				

**Table 3.** Magnetometers parameters resulted from using the entire dataset and RIST in 3-D calibration. (**a**) Scale factor. (**b**) Bias.

(**a**)						
	**X Scale Factor**		**Y Scale Factor**		**Z Scale Factor**	
	**All**	**RIST**	**All**	**RIST**	**All**	**RIST**
**Test1**	1.0371	1.0325	1.1141	1.0985	0.9394	0.9317
**Test2**	1.0479	1.0465	1.1003	1.1014	0.9364	0.9384
**Test3**	1.0153	1.0158	1.0895	1.0860	0.9318	0.9324
**Test4**	0.9136	0.9249	0.9761	0.9718	0.8453	0.8541
**Test5**	0.9286	0.9248	0.9817	0.9859	0.8368	0.8460
**Test6**	0.9454	0.9311	1.0169	1.0167	0.8733	0.8674

(**b**)						
	**X Bias**		**Y Bias**		**Z Bias**	
	**All**	**RIST**	**All**	**RIST**	**All**	**RIST**
**Test1**	38.1795	40.0139	30.8485	38.8051	135.8694	128.0139
**Test2**	36.3021	35.7039	34.2721	33.6313	135.5421	134.6297
**Test3**	40.3624	38.9419	37.8209	36.4251	134.4415	134.2701
**Test4**	28.4165	16.2188	46.2673	53.2383	121.8317	114.8561
**Test5**	34.5628	34.1847	58.8522	58.1043	129.9453	126.9294
**Test6**	46.1353	40.1545	33.8024	37.6382	126.3606	128.5436

**Table 4.** Magnetometers parameters resulted from using the basic PSO and MPSOT in 3-D calibration. (**a**) Scale factor. (**b**) Bias.

(**a**)						
	**X Scale Factor**		**Y Scale Factor**		**Z Scale Factor**	
	**PSO**	**MPSOT**	**PSO**	**MPSOT**	**PSO**	**MPSOT**
**Test1**	1.0267	1.0228	1.1643	1.1334	0.9385	0.9515
**Test2**	1.0485	1.0449	1.0994	1.0998	0.9385	0.9325
**Test3**	1.0079	1.0082	1.0807	1.0903	0.928	0.9371
**Test4**	0.9249	0.9152	0.9717	0.9868	0.8539	0.8452
**Test5**	0.9235	0.9008	0.9812	0.9916	0.8504	0.8608
**Test6**	0.9227	0.9276	1.0191	1.0281	0.8703	0.8644

(**b**)						
	**X Bias**		**Y Bias**		**Z Bias**	
	**PSO**	**MPSOT**	**PSO**	**MPSOT**	**PSO**	**MPSOT**
**Test1**	40.0418	35.2361	8.1353	23.3182	133.0826	150.0231
**Test2**	36.1018	25.7854	33.8402	42.4474	134.9986	130.0379
**Test3**	33.6985	32.2681	32.9084	20.48	135.5549	139.0494
**Test4**	18.5793	8.9173	51.7038	49.8835	115.2107	128.7535
**Test5**	32.2353	33.1784	59.1892	49.9255	127.8947	126.5982
**Test6**	38.1294	36.6339	31.4223	28.0506	130.0527	119.8037
